# CDAP: An Online Package for Evaluation of Complex Detection Methods

**DOI:** 10.1038/s41598-019-49225-7

**Published:** 2019-09-04

**Authors:** Ali M. A. Maddi, Fatemeh Ahmadi Moughari, Mohammad Mehdi Balouchi, Changiz Eslahchi

**Affiliations:** 1 School of biological sciences, Institute for research in fundamental sciences(IPM), Tehran, 193955746 Iran; 2 Department of Computer Sciences, Faculty of Mathematics, Shahid Beheshti University, G.C., Tehran, 1983963113 Iran

**Keywords:** Classification and taxonomy, Computational models, Software

## Abstract

Methods for detecting protein complexes from protein-protein interaction networks are of the most critical computational approaches. Numerous methods have been proposed in this area. Therefore, it is necessary to evaluate them. Various metrics have been proposed in order to compare these methods. Nevertheless, it is essential to define new metrics that evaluate methods both qualitatively and quantitatively. In addition, there is no tool for the comprehensive comparison of such methods. In this paper, a new criterion is introduced that can fully evaluate protein complex detection algorithms. We introduce *CDAP* (Complex Detection Analyzer Package); an online package for comparing protein complex detection methods. CDAP can quickly rank the performance of methods based on previously defined as well as newly introduced criteria in various settings (4 PPI datasets and 3 gold standards). It has the capability of integrating various methods and apply several filterings on the results. CDAP can be easily extended to include new datasets, gold standards, and methods. Furthermore, the user can compare the results of a custom method with the results of existing methods. Thus, the authors of future papers can use CDAP for comparing their method with the previous ones. A case study is done on YGR198W, a well-known protein, and the detected clusters are compared to the known complexes of this protein.

## Introduction

Proteins are known as the smallest biological units and many biological activities are carried out by them. These small factories mainly do their own biological activities in groups which are known as complexes^1^. In other words, a complex is a group of proteins that gather together at a specific time to perform a biological activity collectively. Individual proteins can participate in different protein complexes. Since the proteins carry out many of the biological processes in the form of complexes, study, and analysis of protein complexes is of significant importance. We know that cells of the simplest living creatures consist of thousands of proteins, so millions of different cases for constituent proteins of a complex are possible. Although there are precise experimental methods to verify the existence of a complex, being time-consuming, high cost and a huge number of candidate complexes that should be considered, have made experimental methods practically inefficient^2^. In these situations, it seems that computational methods can be quite effective in limiting probable cases of a complex^3^.

Methods for detecting protein complexes from protein-protein interaction networks (PPINs) are of the most critical computational methods which have been widely used recently. Several methods have been proposed for the discovery of protein-protein interactions in recent years^4^. By using these methods, very large networks of interactions between proteins can be created which are a suitable bed for complex detection methods. These networks are commonly known as PPI networks and can be modeled by weighted graphs; so that each vertex represents a protein and each interaction between two proteins is represented by a weighted edge between the corresponding nodes. Different laboratory methods that detect interactions between proteins have different kinds of errors. Thus, each interaction that is detected using these methods has a different degree of reliability. The difference in the amount of reliability can be modeled by the weight of the associated edge in the mentioned graph^5^.

Complexes can be considered mainly as dense subgraphs of PPINs^6^. In other words, since the interactions within the proteins of a complex with each other (internal interactions) are usually much greater than the interactions of these proteins with other proteins outside the complex (external interactions), complexes can be usually considered as dense subgraphs in PPI network^7,8^. Note that not only should the density be considered in the comparison of the number of internal interactions with external interactions, but also the total weight of internal interactions should be higher than the total weight of external interactions. Many algorithms have been proposed to detect protein complexes using this idea, among which the most important ones are the followings:

- CFinder^9^ forms clusters by a greedy method for finding maximal cliques with at least k vertices.
- ClusterONE^10^ uses an iterative greedy method to detect protein complexes. In each iteration, it selects a vertex as a core and extends it through the neighboring vertices with the aim of increasing the density of the growing cluster.
- RNSC^11^ uses the idea of partitioning the network into subgraphs with the aim of maximizing a cost function. The obtained subgraphs are introduced as candidates for protein complexes.
- IMHRC^12^ removes a part of network hubs from the network. Then, by using a greedy evolutionary method such as ClusterONE, computes the initial clusters. Then, it returns some of the removed hubs to the network and applies a filtering step at the end.
- MCL^4^ utilizes random walk theory and Markov chains rule by iterating two stages of inflation and expansion, which are implemented using matrix multiplication and summation.
- RRW^13^ forms each cluster by selecting its core and adding the closest and most probable vertices via the random walk. The decisions for adding vertices to clusters are taken based on the maximization of flow in clusters.
- ProRank^14^ ranks the proteins based on their importance. The essential proteins have high interaction and evolutionary similarity with the others. After ranking proteins, the complexes are detected using the spoke model.
- ProRank+^15^ has the similar steps to ProRank. But it has several variations. ProRank requires a similarity matrix as an input which indicated the similarities between proteins, while ProRank+ do not need such input. Furthermore, ProRank+ can detect complexes with overlap and has some post-processing steps for refining complexes.
- PEWCC^16^ assesses the reliability of the interaction data, then predicts protein complexes based on the concept of weighted clustering coefficient.

Since there are numerous methods for detecting protein complexes, it is essential to evaluate and compare their results. The inherent properties of complexes, such as the overlapping feature, have led to defining various evaluation metrics for comparing and ranking these algorithms. Although different evaluation criteria have been introduced in recent years, none of them are able to evaluate the results of the algorithms comprehensively and cover their shortcomings. Therefore, introducing a new criterion for analysis methods both quantitatively and qualitatively is requisite. Furthermore, an efficient online package for an extensive comparison of protein complex prediction algorithms was not available.

In this paper, we present a survey of previously defined criteria and scrutinize their drawbacks. Also, we define a new evaluation metric for ranking protein complex detection algorithms.

We introduce “CDAP” an online package for analysis and comparison of such algorithms. “CDAP” ranks algorithms on various PPI datasets (Collins, Gavin, Krogan-core and Krogan-extended) based on three well-known gold standard (MIPS, SGD and CYC2008). It is able to rank methods according to each of the evaluation metrics. The functionality of the package can be easily extended to incorporate new datasets, gold standards and compare novel methods with previously developed methods. Moreover, it visualizes the performance of each method based on evaluation metrics, which can help the user to get a better visual intuition of the performance of methods. Additionally, it has several custom options for integrating and filtering the results of methods.

## Application

The CDAP server can be accessed from http://www.eslahchilab.ir/softwares/cdap. In order to compare the results of protein complex detection algorithms, the user should set up the desired setting in 5 steps.

1. The settings about the PPIN dataset must be specified. The list of PPIN datasets contains Collins, Gavin, Krogan-core, and Krogn-Extended.
2. The gold standard should be selected. The gold standards are Mips, SGD, and CYC2008.
3. The algorithms shall be chosen from the list of {Cfinder, ClusterOne, RNSC, IMHRC, MCL, RRW, ProRank, ProRank+, and PEWCC}. A part of this list is shown in Fig. 1.

**Figure 1:**
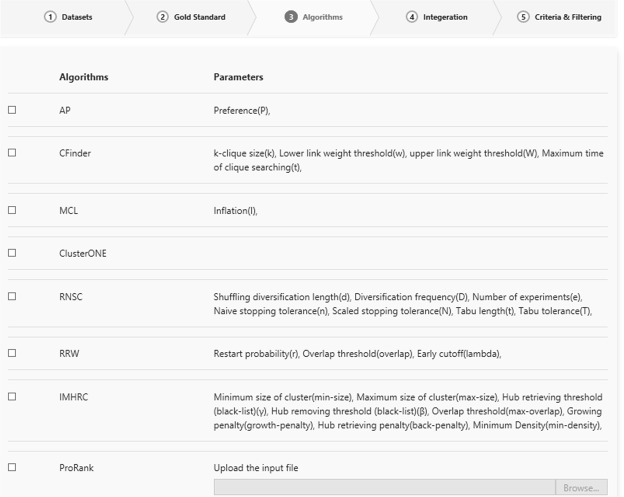
Screenshot from CDAP server, showing the list of algorithms that can be chosen by the user.
4. The user can choose the type of integration, which is optional.
5. At the end, the evaluation criteria should be selected. The sets of evaluation criteria include (*SN*, *PPV and ACC*), (*MMR*), (*Recall*, *Precision* and *F*– *measure*), (*Recall*^+^, *Precision*^+^ and *F* – *measure*^+^), (*Recall*_*N*_, *Precision*_*N*_ and *F* – *measure*_*N*_) and (*AUMF*). There are also some filtering options in this step which are optional.

and Then, he/she may choose the algorithm(s) from the list shown in which are supposed to.

When the user complete these steps, the server applies algorithms on PPIN and evaluate the outputs with the gold standard based on evaluation criteria. Once a method is chosen, the default parameter values are shown in the related boxes; however, the user can change them arbitrarily. It should be noted that the default values of parameters are the values that have been recommended by their authors or by Nepusz *et al*.^10^. In fact, Nepusz *et al*. have calculated the best setting for every algorithm (except IMHRC) on each dataset and MIPS and SGD gold standards. The default values of IMHRC parameters on each dataset with MIPS and SGD gold standards are recommended by Maddi *et al*.^12^. Note that these default parameters are not for CYC2008 since it is less studied. ProRank, is the only method in this package that needs an extra biological information of protein similarities.

### Presentation of results

**Figure 2:**
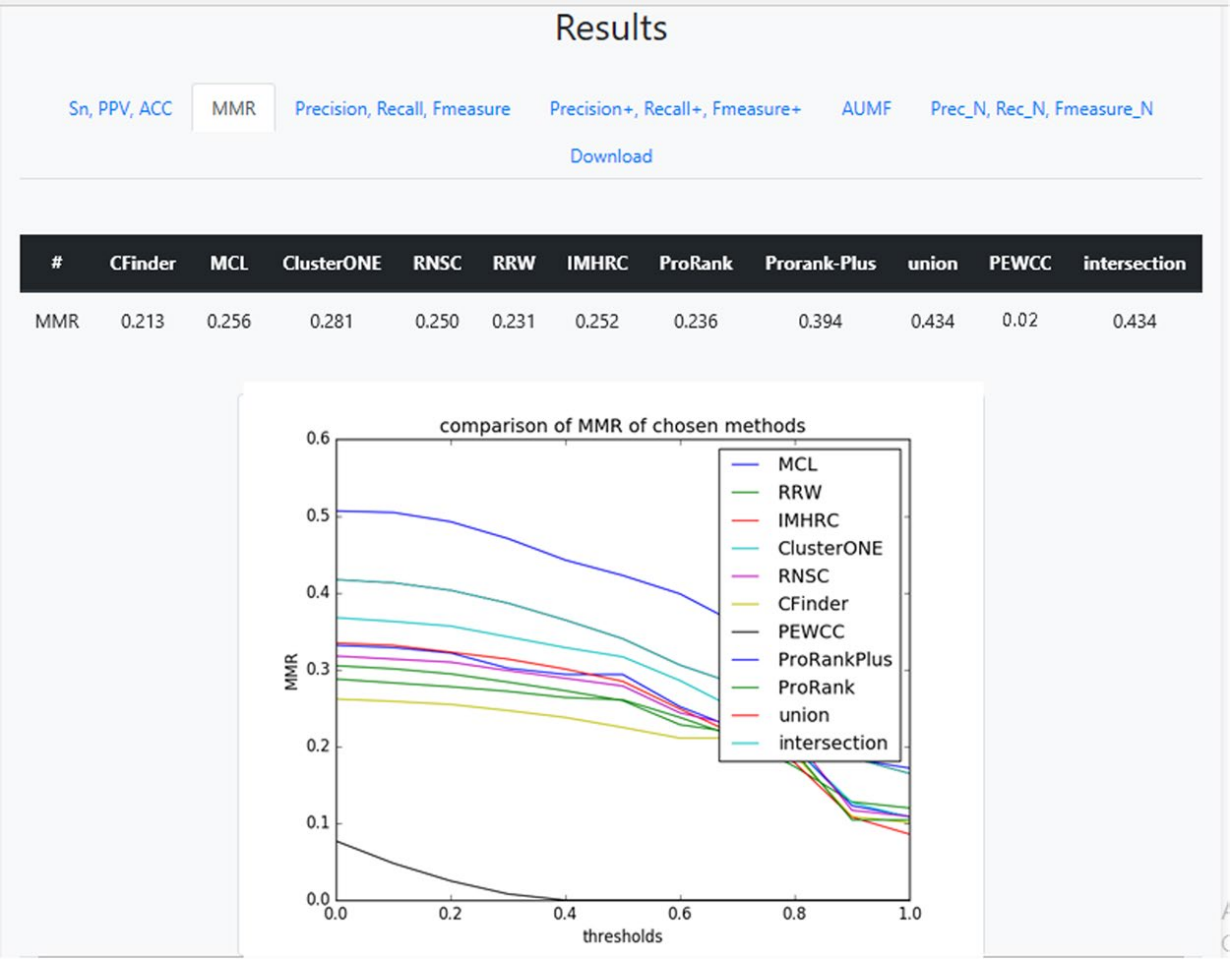
A sample of results of the package.

Detected clusters by the selected algorithm are downloadable from the output section of the server. The computed criteria for each of the algorithms are tabulated and also displayed via multi plots. For each criterion, the comparisons are presented in a tab that contains a multiplot and a table. The multiplot shows the computed criteria of all selected algorithms indexed by various thresholds used in the computation of criteria. The areas under these curves are projected in a table. An example of outputs is shown in Fig. 2. In case of selecting (*SN*, *PPV* and *ACC*) or (*AUMF*) sets of criteria, no plot is shown, since setting a threshold for computing these criteria is meaningless. Thus, their absolute values are reported in a table.

### Custom Options

In addition to the listed algorithms, the user may submit the detected clusters obtained by any method. It should be noted that when the results of a (or several) new method(s) are uploaded, the selected dataset should be the one that is used the process of obtaining the results. Moreover, this server has the ability to run the mentioned algorithms on other PPIN datasets. So the user can upload custom datasets. It is also possible for the user to upload new gold standards for conducting comparison based on different complexes. The valid formats for uploading custom algorithms, PPINs and gold standards are described in Supplementary Table 2. Additionally, one can extend the server with a new criterion, protein complex detection algorithm, PPIN or gold standard, in such a way that future users can make use of them, by sending them to the contact mail to be adjusted and added to the server. The guidelines are represented in Supplementary Table 3. It is also possible to set a threshold *θ* for computing criteria based on it. To be more accurate, a cluster will be considered to be similar to a gold standard complex, if their overlap is greater than this threshold.

Moreover, the package has some option for filtering the detected clusters. The user can specify the minimum and maximum size of detected protein clusters. On the top of that, a very practical option of this package is that user can enter the STRING ID of a protein in order to receive all clusters detected by each method that contains the specified protein, an example of such applicable option is presented in the following. Another type of filtering can be done based on reliability, such that the results of methods will be filtered with the clusters that are detected by at least *β* methods.

### Integration

The package has the capability to integrate the methods’ results. It can yield the intersection or union of the clusters detected by various methods. To do this, it constructs a k-partite graph (*k* is the number of selected methods). Each part contains the nodes that are representatives of its clusters. There will be an edge between two nodes of two different parts if their corresponding clusters have more than $\varphi  \%$ overlap. Then, all maximal cliques with size greater than $\psi$ are considered and the clusters are used for intersection or union. So, the integration of results will contain the clusters with high reliability that are detected (with some alterations) by most methods. $\varphi$ and $\psi$ can be specified by user, otherwise their default values are considered which are 0.5 and *k*/2, respectively. Once the union or intersection is selected, the computed criteria for the integrated results are presented in the Results tab as shown in Fig. 2 and its file can be downloaded.

### An example of Integration

Integrating the results of several methods may yield better results. As an example, we selected all methods and integrate them with different values of hyperparameters $\varphi$ and $\psi$. The best results were obtained in two cases:

- Integration11: $$\varphi =1$$ and $$\psi =1$$
- Integration12: $$\varphi =1$$ and $$\psi =2$$

ProRank is excluded from this analysis, because it uses extra biological information beside topology, while other methods are use only topological data. Thus, integrating ProRank with topological-based method is not suitable. It should be mentioned that when $\varphi =1$, union and intersection yield the same results. The criteria value for Integration11, Integration12 and other methods are presented in Table 1. It can be seen that integration can improve the results of almost all methods. Integration11 improves all methods in terms of *MMR*, *Sn*, *ACC*, *Recall*, *Recall*^+^, and *Precision*_*N*_. Moreover, Integration12 improves all methods in terms of *MMR*, *AUMF*, *Recall*, and *Recall*_*N*_.

**Table 1:** The comparison of integrated results and the results of single methods.

Method:	*MMR*	*Sn*	*PPV*	*ACC*	*AUMF*	*Prc*	*Rec*	*F*	Prc*	*Rec* ^+^	*F* ^+^	*Prc*_*N*_	*Rec*_*N*_
Integration11	**0.444**	**0.705**	0.372	**0.522**	0.518	0.067	**0.627**	0.083	0.039	**0.595**	0.074	**0.461**	0.179
Integration12	**0.323**	0.548	0.397	0.466	**0.667**	0.321	**0.526**	0.395	0.286	0.430	0.344	0.336	**0.417**
MCL	0.256	0.571	0.415	0.487	0.601	0.459	0.493	0.494	0.351	0.339	0.345	0.332	0.328
RRW	0.231	0.459	0.385	0.420	0.572	0.452	0.454	0.453	0.409	0.292	0.341	0.278	0.408
RNSC	0.250	0.545	0.405	0.470	0.616	0.485	0.487	0.486	0.420	0.324	0.366	0.321	0.369
IMHRC	0.252	0.589	0.400	0.486	0.595	0.495	0.495	0.495	0.343	0.343	0.343	0.336	0.296
ClusterONE	0.281	0.640	0.416	0.516	0.599	0.378	0.525	0.437	0.276	0.376	0.319	0.364	0.249
CFinder	0.213	0.669	0.309	0.455	0.548	0.407	0.416	0.412	0.449	0.268	0.336	0.285	0.236
ProRank+	0.236	0.665	0.406	0.520	0.575	0.096	0.579	0.133	0.109	0.520	0.181	0.430	0.204
PEWCC	0.012	0.461	0.326	0.338	0.030	0.051	0.085	0.053	0.010	0.082	0.018	0.236	0.038

Criteria names are abbreviated: (*Prc* stands for *Precision*, *Rec* stands for *Recall*, *F* stands for *F* – *measure*). The criteria values that are improved by integration, are represented in Bold.

### An example of filtering by reliability

As an example of the package capability of filtering method results by reliability, we applied this filtering on ClusterOne method with $\beta =3$. The criteria values are presented in Table 2. It can been seen that this filtering improved the values of *Precision*, *F* – *measure*, *Precision*^+^, *F* – *measure*^+^, and *Recall*_*N*_. It should be noted that the filtering may decrease the quality of performance in terms of some criteria, and it highly depends on the value of hyperparameters.

**Table 2:** The comparison of filtered results and the original results of ClusterOne method.

Method:	*MMR*	*Sn*	*PPV*	*ACC*	*AUMF*	*Prc*	*Rec*	*F*	*Prc* ^+^	*Rec* ^+^	*F* ^+^	*Prc*_*N*_	*Rec*_*N*_
Original	0.281	0.640	0.416	0.516	0.599	0.378	0.529	0.439	0.276	0.376	0.319	0.364	0.249
Filtered	0.198	0.624	0.399	0.499	0.528	**0.454**	0.449	**0.452**	**0.363**	0.302	**0.330**	0.329	**0.268**

Criteria names are abbreviated: (*Prc* stands for *Precision*, *Rec* stands for *Recall*, *F* stands for *F* – *measure*). The criteria values that are improved by integration, are represented in Bold.

### Complexes of YGR198W: An example of filtering by protein ID

YGR198W/YPP1 is an essential protein in Saccharomyces Cerevisiae. It also performs fundamental functions in Human, Mouse and other species. It is a Cargo-transport protein involved in endocytosis. Furthermore, it plays role in the assembly and recruitment of multiple copies of the kinase into phosphoinositide kinase (PIK) patches at the plasma membrane^17–21^.

Knowing the complexes that included this protein, helps us to have better insight into its functionality. Four complexes have been reported for this protein in Yeast Resource Center (YRC) and Database of Interacting Proteins (DIP) which are listed in Supplementary Table 4. CDAP can help us to retrieve all detected clusters including this protein quickly using its filtering by protein ID option. It should be noted that this protein is included just in Krogan-core and Krogan-extended PPI datasets. Using CDAP, we executed all methods on these two datasets based on both gold standards.

Among all methods, just Cfinder, RNSC, and MCL returned clusters that contain YGR198W. Each of these methods on each setting found at most one cluster containing YGR198W, but with different size. As it is shown in Table 3, eight clusters were detected when YGR198W was provided as a query, 3 of which share common proteins with reported complexes in YRC; 2 of these clusters are detected by MCL and another cluster is detected by RNSC. It should be noted that both clusters detected by MCL are verified with the same complex, i.e. there is a complex reported in YRC that all its proteins are detected by MCL, but in the second cluster, MCL identified more proteins as the constituent. Five other clusters have not been documented in earlier studies as known complexes of YGR198W. Such clusters may give clue to discovering other complexes of this protein in further studies. The detected clusters are presented in Supplementary Table 5.

**Table 3:** Number of verified proteins in each detected cluster.

Dataset:	Krogan-Core		Krogan-Extended	
Gold Standard	MIPS	SGD	MIPS	SGD
CFinder	1 of 8	1 of 8	0 of 0	0 of 0
MCL	**15 of 15**	0 of 0	**14 of 22**	1 of 2
RNSC	1 of 2	1 of 2	**2 of 3**	0 of 0

To clarify the notation, *x* of *y* means that the method finds a cluster containing YGR198W with *y* proteins and there exists a known complex that shares *x* protein in common with the detected cluster.

## Discussion

In our survey of previously defined criteria, it has alluded that some of the criteria values are dependent to threshold *θ*. The dependency of criteria to the value of *θ* has several drawbacks.

- By changing the values of threshold *θ*, the meaning of “true” and “false” for detected clusters is altered.
- Setting an appropriate value for *θ* is crucial, challenging and hard.
- For a specified value of *θ*, the amounts of the difference of edge weights to *θ* are not considered. If two methods have the same bipartite graph that all weights are greater than *θ* and in the second graph, all edge weights are duplicated. However these two methods do not have equal performance, the calculated values of criteria for these two methods are equal; since in the calculation of threshold-based criteria, the quantities of weights are ignored.

There is a critical point that should be noticed in utilizing threshold dependent criteria that is by changing the value of the threshold, a ranking of method performances will be altered. Therefore, for a specified evaluation metric and the specified value of the threshold, method A may be better than method B; while with a different value of the threshold and the same criterion, method B is better than method A.

In previous papers such as^9,12^ evaluations and validations are based on a specific value of the threshold, which is not an appropriate assessment. For example, RNSC is reported in^12^ as a method that does not perform well, while this conclusion is based on a specific value of the threshold. Using CDAP, one can realize that RNSC is of the best methods based on the area under curves of the criteria that are listed in Table 4. To overcome this problem, we have used the area under the curve for each threshold dependent criterion. In fact, in CDAP, when the user selects a criterion, the value shown in the output table is the area under the curve of the values of this criterion indexed by values of the threshold.

**Table 4:** A summary of calculated values of area under curves of the criteria using CDAP.

Criteria	*ACC*	*MMR*	*Fmeasure*	*Fmeasure* ^+^	*AUMF*	*Precision*_*N*_	*Recall*_*N*_
CFinder	0.465	0.207	0.362	0.281	0.488	0.289	0.212
MCL	0.457	0.208	0.340	0.255	0.463	0.272	0.223
ClusterOne	0.469	0.242	0.293	0.251	0.493	0.315	0.218
RNSC	0.451	0.239	0.422	0.308	0.547	0.304	0.291
RRW	0.418	0.217	0.430	0.319	0.536	0.250	0.375
IMHRC	0.421	0.244	0.334	0.292	0.536	0.271	0.303
ProRank	0.445	0.217	0.447	0.297	0.514	0.291	0.275
ProRank+	0.466	0.327	0.147	0.196	0.523	0.378	0.295
PEWCC	0.464	0.036	0.056	0.039	0.075	0.315	0.074

In addition to the definition of AUMF which is not dependent on the threshold, we revise previous criteria by considering the area under curve concept. For instance, for *F* – *measure* criterion, the area under the curve of the graph which plots the *F* – *measure* vs. threshold on x- and y-axes is calculated. It is worth mentioning that CDAP can compute the value of threshold dependent criteria for a particular threshold in case of user demand. Furthermore, CDAP computes two new values, namely “*AUPR*” and “*AUPR*^+^”. “*AUPR*” is the area under curve of *Precision* vs. *Recall* and “*AUPR*^+^” is the area under curve of *Precision*^+^ vs. *Recall*^+^.

## Conclusion

Methods for detecting protein complexes from protein-protein interaction networks (PPIs) are one of the most critical computational methods which have been widely used recently. Several methods have been proposed for the discovery of protein-protein interactions in recent years, namely Cfinder, MCL, RNSC, RRW, ClusterOne, IMHRC, ProRank, ProRank+, and PEWCC. Evaluating these methods is of high importance. The inherent properties of complexes, such as the overlapping feature, have led to defining various evaluation metrics and criteria for comparing and ranking these algorithms. Many evaluation metrics have been proposed previously such as *ACC*, *PPV*, *SN*, *Precision*, *Recall*, *F* – *measure*, *Precision*_*N*_, *Recall*_*N*_, and *MMR*, each of which has some drawbacks and cannot fully reflect the quality of an algorithm. We analyzed these evaluation metrics in this paper and expressed their flaws by presenting some examples. It is reasonable that a protein complex detecting algorithm performs well whenever it returns the clusters the same as the complexes in the gold standard. So, the evaluation metrics should be able to measure the quality and quantity of one-to-one and spanning relationship between the estimated clusters and complexes in the gold standard. In this paper, we introduced a new evaluation metric *MMR* + *Fmeasure*^+^. The newly introduced metric can better express the quality and quantity of the one-to-one and spanning relationship between the clusters and complexes. Nevertheless, its value depends on the value of the threshold. Thus, we defined a new criterion that is invariant with respect to values of threshold, which is the Area Under (*MMR* + *Fmeasure*^+^, *θ*) curve (*AUMF*). This criterion has the feature of assessing methods both qualitatively and quantitatively in addition to covering the drawbacks of previous criteria.

We have developed CDAP, an online package for the comparison and visualization of the performance of protein complex detection methods. It is available at http://www.eslahchilab.ir/softwares/cdap. CDAP lets the user select the PPI dataset, gold standard, protein complex detection method and set of criteria for evaluation.

Moreover, we revised previously defined criteria that are dependent on the threshold of *θ*. In CDAP the calculated value for such criteria is the area under curves of criteria indexed by values of *θ*.

CDAP also has the ability to compare the results of any custom method when its outputs are uploaded in a valid format on the website. Furthermore, one can upload new datasets or gold standards for training and evaluating algorithms. We tried to include AP^22^ and CMC^7^ methods in our package, but there was some problem in the compatibility of platforms that we are going to remove them. We are trying our best to add these methods and other newly proposed methods to our package.

One of the most applicable queries that can be done by CDAP is the option of filtering detected clusters by setting minimum or maximum size of their proteins or by specifying a protein as a query, which in this case the CDAP find all clusters detected by methods that contain the specified protein. Another type of filtering is based on the reliability such that filtered results contain only the clusters that are detected by several methods. A useful capability of the package is the integration of methods’ results via intersection and union.

Due to the rapid trend of producing and analyzing data, proposing novel algorithms and defining new criteria, anyone can extend CDAP simply by sending new datasets, gold standards, methods or criteria to the contact mail. The guidelines for sending files is described in Supplementary Table 3.

To sum up, CDAP can be a very useful package to compare methods exhaustively and comprehensively and can facilitate the validation process for authors of future papers and help them in comparison of their proposed method to the previous ones.

## Methods

### Datasets

The datasets of PPINs used in this package are: Collins^5^, Gavin^1^, Krogan-core^23^ and Krogan-extended^23^. These datasets were used to learn methods in the package. All settings and parameters in every dataset were set based on what the original papers have proposed. We removed self-interactions and isolated proteins from all datasets. The weights of PPIs in the Gavin dataset are Socio-affinity indices, which show the log-odds of number of times that pairs of proteins are observed together as preys, or bait and a prey^24^. Selection of PPIs from the Collins was based on purification enrichment score which contains the top 9074 interactions. The weights of all PPIs in Krogan-core are greater than 0.273, while the weights of all PPIs in Krogan-extended are greater than 0.101.

### Gold standards

The results were compared with three gold standards for Saccharomyces cerevisiae, namely SGD, MIPS and CYC2008:

- Gene Ontology-based protein complex annotations from SGD (Saccharomyces Genome Database)^25^. SGD includes Gene Ontology (GO) annotations which provide useful biological information for producing reference complexes.
- The catalog of protein complexes from MIPS^26^ (the Munich Information Center for Protein Sequences). The MIPS catalog has a hierarchical structure so the complexes may be composed of several subcomplexes^10^.
- The benchmark protein dataset CYC2008 that contains manually curated heteromeric protein complexes^27^.

### Previously introduced evaluation metrics

**Figure 3:**
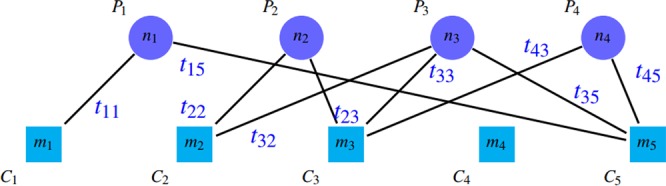
A schematic representation of relations between detected clusters and gold standard complexes.

A common approach for comparing and evaluating protein complex detection algorithms is to match their outputs with reference complexes in gold standard sets. We call the true complexes of the gold standard as “reference complex” and detected complexes by methods as “detected clusters”. A remarkable point is the existing overlap among protein groups. These overlaps between reference complexes, as well as the existence of similar overlap between the protein clusters detected by the above algorithms, have led to different comparison and evaluation methods. It should be noted that a reference protein complex may correspond to several clusters detected by the algorithms. Conversely, a detected cluster may correspond to more than one reference protein complex. It is worth mentioning that the relation between detected clusters and gold standard complexes is often not complete and is sometimes partial. Thus, some reference complexes may have no relation with any detected clusters and vice versa. These connections can be represented by a bipartite graph, in which the circle nodes (*P*_*i*_) in the first part are representatives of gold standard complexes and the square nodes (*C*_*j*_) in the second part are the representatives of detected clusters. Figure 3 shows a schematic illustration of such relations. The number on each node (*n*_*i*_ or *m*_*j*_) shows the number of proteins in that node (cluster or complex). The weight *t*_*ij*_ on edge (*P*_*i*_, *C*_*j*_) reports the number of common proteins between complex *P*_*i*_ and cluster *C*_*j*_.

Undoubtedly, these relations must be measured quantitatively and qualitatively. In other words, not only the number of unique matches among the members of the two groups should be taken into account, but also the quality of each matching is very important and should be considered. Three common categories of criteria are introduced in articles for such evaluation. We survey evaluation metrics in two parts: qualitative metrics and quantitative metrics.

*Qualitative metrics*

**Figure 4:**
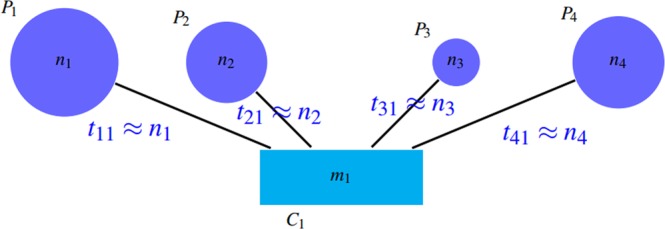
A giant component is among detected clusters. So, $Sn\approx \tfrac{{\sum }_{i=1}^{n}\,{n}_{i}}{{\sum }_{i=1}^{n}\,{n}_{i}}\approx 1$ and $PPV=\tfrac{{n}_{1}}{{n}_{1}+{n}_{2}+{n}_{3}+{n}_{4}}$.
   Additionally, if there are a lot of similar groups between the detected clusters, the value of this criterion is almost equal to value of *SN* after removing redundant clusters (removing all similar groups except one of them). Although, PPV tends to 1 by repeating similar clusters, while the results of algorithms is not great. This issue can be seen in Figs 5 and 6.

- *SN*, *PPV* and *ACC*
  The first category consists of sensitivity (*SN*), positive predictive value (*PPV*), and accuracy (*ACC*) introduced by Brohee and van Helden^28^. *ACC* is the geometric mean of two criteria: *SN* and *PPV*. Suppose that there are *n* complexes in a gold standard as the member of the reference complex and *m* clusters are detected by an algorithm, *t*_*ij*_ represents the number of proteins that the reference complex *i* and the detected cluster *j* share in common and *n*_*i*_ denotes the number of proteins that are in the reference complex i. *SN* and *PPV* are defined as follows:
  1 $$PPV=\frac{{\sum }_{j=1}^{m}\,{{\rm{\max }}}_{i}{t}_{ij}}{{\sum }_{i=1}^{n}\,{\sum }_{j=1}^{m}\,{t}_{ij}}$$
  2 $$SN=\frac{{\sum }_{i=1}^{n}\,{{\rm{\max }}}_{j}{t}_{ij}}{{\sum }_{i=1}^{n}\,{n}_{i}}$$
  It is evident that the values of *SN* and *PPV* is in range $[0,1]$. The expected value of *PPV* is low; since its numerator is the sum of *m* elements and its denominator is the sum of *nm* elements. All criteria of this category focus on the quality of detected clusters. In other words, they measure the number of proteins that reference complexes and detected clusters share in common. According to the definitions, *SN* represents the ratio of proteins in reference complexes that are corresponded to the detected clusters. Ignorance of the impacts of giant components and redundant groups in detected clusters is one of the shortcomings of this benchmark. Suppose that there is a giant component among the detected clusters that contains all the proteins in the reference complexes. It is obvious that the value of *SN* criterion will be maximized in that case. Figure 4 shows this issue.

**Figure 5:**
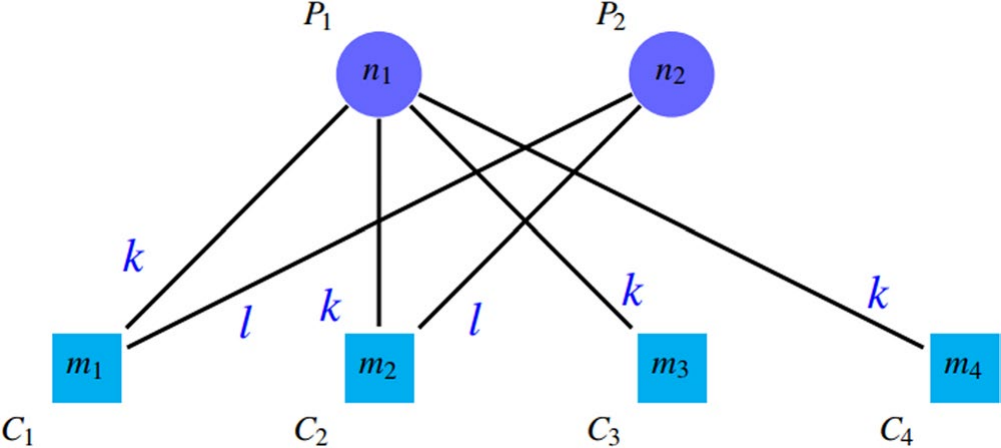
Four clusters in this figure, are so similar; so $l < k$, $SN\approx (k+l)/({n}_{1}+{n}_{2})$ and $PPV=4k/(4k+2l)$. PPV tends to 1 by adding similar clusters.

**Figure 6:**
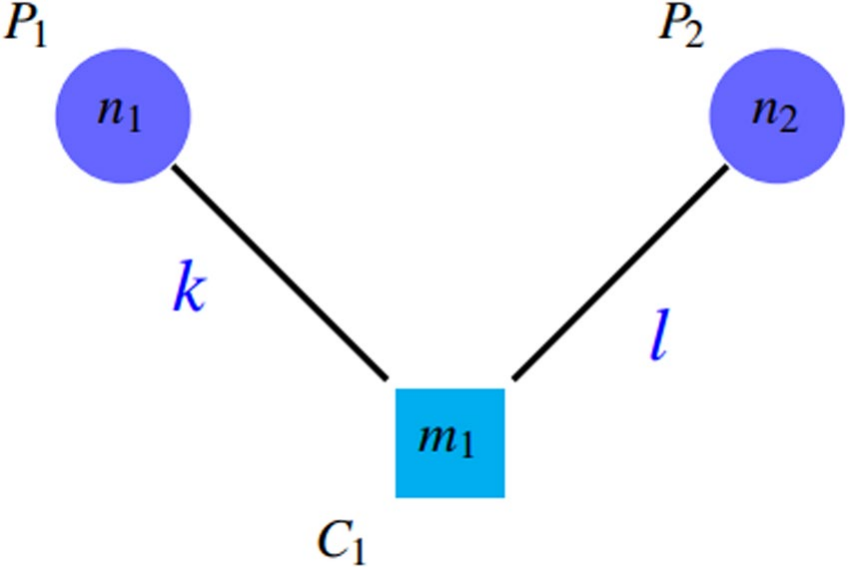
This graph is the same as previous figure, except that similar clusters are removed. The value of *SN* is not changed after removing similar clusters but *PPV* decreased ($k > l$). $SN\approx (k+l)/({n}_{1}+{n}_{2})$ and $PPV=k/(k+l)$.
  *PPV* is defined in such a way that the accumulation of proteins in a group (the existence of a giant component) leads to reduction of *PPV* by incresing the denominator. Notice to *PPV* values in Fig. 4. Unfortunately, *PPV* ignores false negatives, i.e. if there are so many complexes that are not matched by clusters well, this issue do not decrease *PPV* value, but it declines *SN* value. Figures 7 and 8 shows this problem.

**Figure 7:**
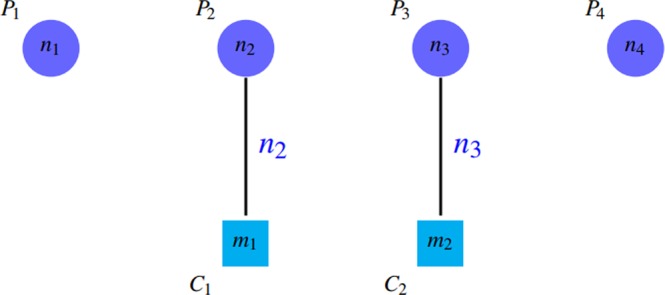
Some complexes are not covered well that are known as false negatives. $SN=({n}_{2}+{n}_{3})/({n}_{1}+{n}_{2}+{n}_{3}+{n}_{4}) < 1$ and $PPV=({n}_{1}+{n}_{2})/({n}_{1}+{n}_{2})=1$.

**Figure 8:**
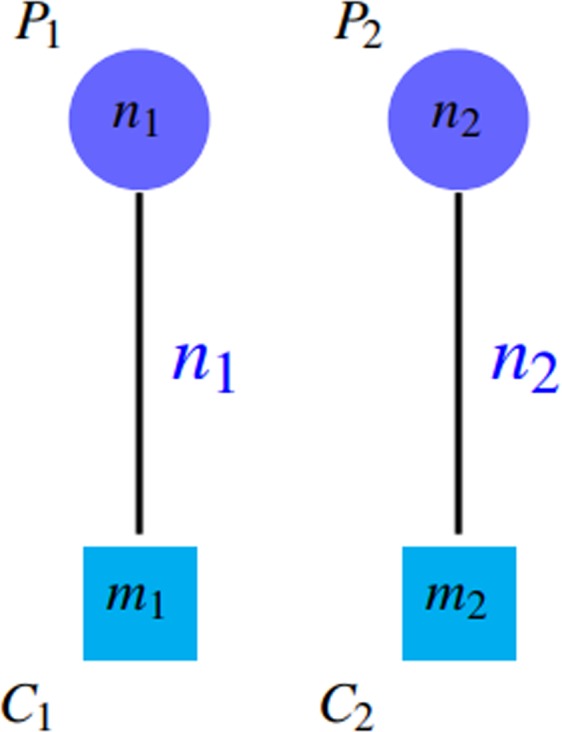
This graph includes the same nodes as the previous figure but not the false negatives. The *SN* value in the previous figure is less than this one due to the false negatives, but the *PPV* values are the same. $SN=({n}_{1}+{n}_{2})/({n}_{1}+{n}_{2})=1$ and $PPV=({n}_{1}+{n}_{2})/({n}_{1}+{n}_{2})=1$.
  Therefore, both of these criteria has some drawbacks. So the accuracy criterion (*ACC*) is used to balance the values of the two criteria of *SN* and *PPV*.
  3 $$ACC=\sqrt{SN\times PPV}$$
  However, *ACC* cannot be considered as a flawless benchmark for evaluating and comparing protein complex detection algorithms. As mentioned above, in case of adding redundant clusters to the results, which leads to low performance of algorithm, *SN* does not change and *PPV* increases, so *ACC* ascends.
  Furthermore, consider a perfect complex detection algorithm that its outputs are exactly the same as the reference complexes in a gold standard. We expect that the criteria values to be the maximum. It is evident that the detected clusters may have some overlaps. For such results, *SN* value will be maximized, while this is not the case for *PPV*. Thus, *ACC* value of this perfect method is not 1. In other words, due to overlaps between the reference complexes, the value of ${t}_{i\ast }$, which is defined as follows, will be greater than *n*_*i*_.
  $${t}_{i\ast }=\mathop{\sum }\limits_{j=1}^{n}\,{t}_{ij}$$
  One can conclude from the equivalence of detected clusters and reference complexes that:
  $$\mathop{{\rm{\max }}}\limits_{j}\,{t}_{ij}=\mathop{{\rm{\max }}}\limits_{i}\,{t}_{ij}={n}_{i}$$
  In this case, *PPV* and *SN* are:
  $$PPV=\frac{{\sum }_{i=1}^{n}\,{n}_{i}}{{\sum }_{i=1}^{n}\,{t}_{i\ast }} < 1,\,SN=1$$
  In fact, due to the overlap between the reference protein complexes, there exist some proteins that belong to more than one protein complexes. Therefore, the numerator of *PPV* formula is almost always smaller than its denominator, so the *PPV* value will never be maximized. This issue is illustrated in Fig. 9. As a result, since we know that the existence of overlap is an intrinsic property of the protein groups, *PPV*, *SN*, and *ACC* criteria are not appropriate metrics.

**Figure 9:**
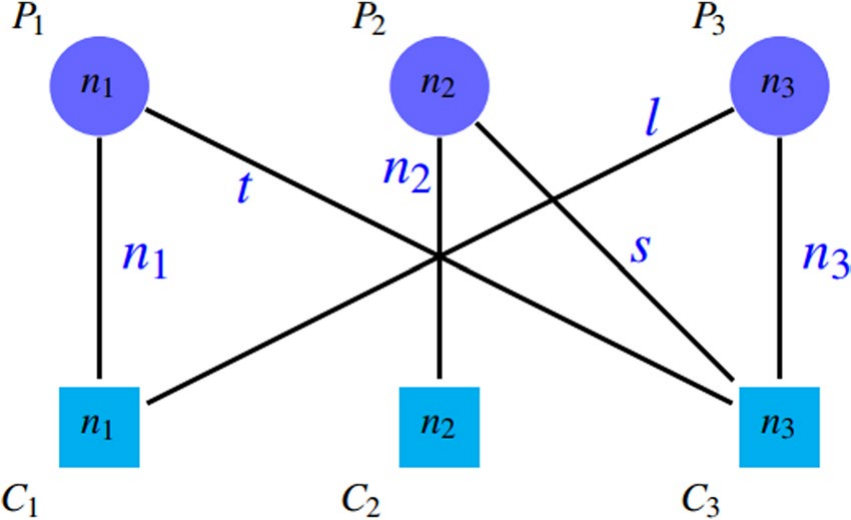
Complexes *P*_1_, *P*_3_ have some overlap and $s < {n}_{2}$, $t < {n}_{1}$, $l < {n}_{3}$. $SN=({n}_{1}+{n}_{2}+{n}_{3})/({n}_{1}+{n}_{2}+{n}_{3})=1$ and $PPV=({n}_{1}+{n}_{2}+{n}_{3})/({n}_{1}+{n}_{2}+{n}_{3}+l+t) < 1$.
  Another important problem is the ignorance of false positives by this category of criteria. This means that detection of any number of incorrect protein clusters have no effect on the values of the criteria in this category. Thus, methods that detect a huge number of clusters (containing a lot of incorrect clusters) often acquire high values of *ACC*. Figure 10 shows this drawback.

**Figure 10:**
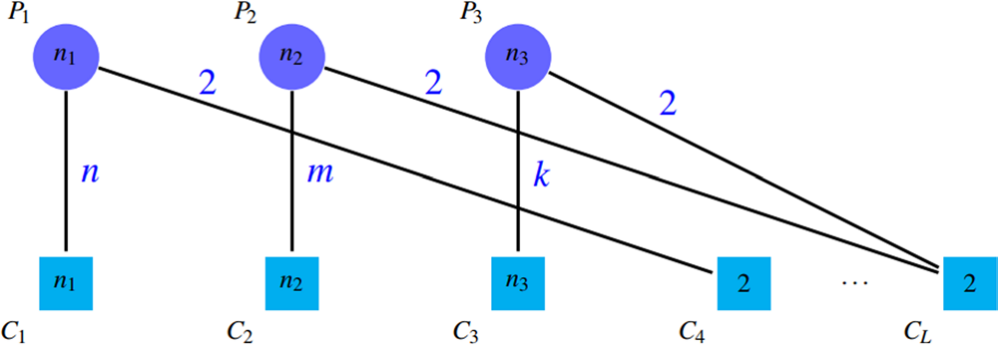
Despite of the existence of many spurious detected clusters, the value of these criteria are high; *n*_1_, *n*_2_, $${n}_{3} > 2$$, $$SN=({n}_{1}+{n}_{2}+{n}_{3})/({n}_{1}+{n}_{2}+{n}_{3})=1$$ and $$PPV=({n}_{1}+{n}_{2}+{n}_{3}+2+\cdots +2)$$/$$({n}_{1}+{n}_{2}+{n}_{3}+$$$$2+\cdots +2)=1$$ and $$ACC=1$$.
  In sum, false positives does not affect *PPV* and *SN*. And False negatives does not affect *PPV*, but they decline *SN*. Therefore, it is essential to analyze methods with another criterion.
- *Pre*_*N*_, *Rec*_*N*_, *F* – *measure*_*N*_
  Zaki *et al*.^14^ defined a set of criteria which is almost analogous to the previous set (*PPV*, *Sn*, *ACC*):
  4 $$Pre{c}_{N}=\frac{{\Sigma }_{i=1}^{|{\mathscr{P}}|}\,|{K}_{i}|}{{\Sigma }_{i=1}^{|{\mathscr{P}}|}\,|{P}_{i}|,\,|{K}_{i}|=ma{x}_{{C}_{j},JCC({P}_{i},{C}_{j}) > \theta }|{P}_{i}\cap {C}_{j}|}$$
  5 $$Re{c}_{N}=\frac{{\Sigma }_{i=1}^{|{\mathscr{C}}|}\,|{K}_{i}|}{{\Sigma }_{i=1}^{|{\mathscr{C}}|}\,|{C}_{i}|,\,|{K}_{i}|=ma{x}_{{P}_{j},JCC({P}_{i},{C}_{j}) > \theta }|{P}_{i}\cap {C}_{j}|}$$
  6 $$F-measure=\frac{2\times Pre{c}_{N}\times Re{c}_{N}}{Pre{c}_{N}+Re{c}_{N}}$$
  where $JCC(P,C)=\frac{|P\cap C|}{|P\cup C|}$ is the Jaccard index. This set of criteria have the same deficiencies as the previous set of criteria (*PPV*, *Sn*, *ACC*). The threshold-dependency of is an extra disadvantage of this set of criteria.
- *MMR*
  *MMR* (Maximal Marginal Relevance) criterion was introduced in^10^ to overcome some of mentioned drawbacks of previous criteria. In this criterion, a complete bipartite weighted graph is constructed based on threshold *θ* similar to the mentioned graph where
  7 $${t}_{ij}=\{\begin{array}{ll}NA({P}_{i},{C}_{j}) & NA({P}_{i},{C}_{j})\ge \theta \\ 0 & ({P}_{i},{C}_{j}) < \theta \end{array}$$
  and
  8 $$NA({P}_{i},{C}_{j})=\frac{|{P}_{i}\cap {C}_{j}{|}^{2}}{|{P}_{i}||{C}_{j}|}$$
  In this definition, *NA*(*P*_*i*_, *C*_*j*_), which is known as neighborhood affinity score, shows how the reference complex *P*_*i*_ has been able to match the detected cluster *C*_*j*_^3^.
  By applying a weighted bipartite graph matching algorithm on this graph, a one-to-one mapping between the first and second sections is obtained. The *MMR* value is calculated by normalizing the total weight of the maximal matching edges on the number of reference complexes, so its value is in range $[0,1]$. The defect of this criteria is that it ignores false positives; i.e. the detection of many spurious clusters do not affect the value of *MMR*. Because adding similar clusters to a set of detected clusters do not change the maximum matching. Thus, both the numerator and denominator do not change. Dependency of this criterion to the value of *θ* is its another disadvantage.
*Quantitative metrics*
- *Precision*, *Recall* and *F* – *measure*
  This category of criteria includes *F* – *measure*, *Precision* and *Recall*. *F* – *measure* is the harmonic mean of *Precision* and *Recall* criteria. These criteria are defined as follows:
  9 $${N}_{p}=|\{{P}_{i}|\,\exists \,{C}_{j},\,NA({P}_{i},{C}_{j})\ge \theta \}|$$
  10 $${N}_{c}=|\{{C}_{j}|\,\exists \,{P}_{i},\,NA({P}_{i},{C}_{j})\ge \theta \}|$$
  11 $$Precision=\frac{{N}_{p}}{|{\mathscr{P}}|}$$
  12 $$Recall=\frac{{N}_{c}}{|{\mathscr{C}}|}$$
  13 $$F-measure=\frac{2\times Precision\times Recall}{Precision+Recall}$$
  where ${\mathscr{P}}$ and ${\mathscr{C}}$ are representatives of the sets of reference complexes and detected clusters, respectively. Zaki *et al*. used slightly different versions of *Precision* and *Recall* as follows^14^:
  14 $${N}_{p}=|\{{P}_{i}|\,\exists \,{C}_{j},\,JCC({P}_{i},{C}_{j})\ge \theta \}|$$
  15 $${N}_{c}=|\{{C}_{j}|\,\exists \,{P}_{i},\,JCC({P}_{i},{C}_{j})\ge \theta \}|$$
  16 $$Pre{c}_{c}=\frac{{N}_{p}}{|{\mathscr{P}}|}$$
  17 $$Re{c}_{c}=\frac{{N}_{c}}{|{\mathscr{C}}|}$$
  Since *Prec*_*c*_ and *Rec*_*c*_ have almost similar meaning to *Precision* and *Recall*, we do not incorporate *Prec*_*c*_ and *Rec*_*c*_ in CDAP.
  Both of these category of criteria attempt to examine the results of protein complex detection algorithms quantitatively. In fact, in these set of criteria, the main attention is to the number of detected clusters and less attention is paid to the internal quality of these clusters. Thus, the matching size more than a specified threshold cannot influence the quality measured by these criteria. This category has several drawbacks:
- The existence of exorbitant overlapping complexes does not have a significant effect on quality reduction. In other words, the low values for *θ* threshold increase the error rate when the size of the groups get bigger. For example, if $\theta =0.25$, for confirmation of a 10-member cluster, it is sufficient that half of its proteins exist in another 10-member gold standard complex. Thereby, the mentioned algorithm can detect similar groups with the mentioned core, all of which have 5 members in common with the gold standard, or detect some redundant groups. Thus, all redundant groups are validated. Although *Recall* does not change but *Precision* increases which lead to an increase in *F* – *measure*. The reason for this drawbacks is that several clusters can be validated by one complex
- The values of the mentioned criteria is dependent on the value of *θ*.

In order to address this issue, we introduce a new set of criteria.

### A new set of evaluation metrics

As mentioned in the previous section, each set of criteria has some drawbacks. One way to have a more expressive set of criteria is to gather various criteria. In order to cover *MMR* shortcomings, *ACC* and *Recall* have been used in^2^. As explained above, *ACC* has a fundamental defect due to the presence of overlap between groups. In addition, *ACC* measures the groups qualitatively similar to *MMR*. Thus, there is no need for *ACC* criterion while using *MMR*. Moreover, usage of *Recall* criterion solely and without *Precision* criterion does not make sense. Therefore, the use of *F* – *measure* instead of the *Recall* criterion is more logical. One can add quantitative evaluation to the main evaluation process by using *F* – *measure*; however, all the deficiencies have not been resolved yet due to the mentioned defects for the second category. We used *MMR* criterion alongside the modified *F* – *measure* to have a more accurate evaluation system. The modified *F* – *measure*, *Precision*, and *Recall* are represented by *F* – *measure*^+^, *Recall*^+^, and *Precision*^+^, respectively.

18 $${N}_{p}^{+}=|\{{P}_{i}|\,\exists \,{C}_{j},\,NA({P}_{i},{C}_{j})\ge \theta ,\,({P}_{i},{C}_{j})\in Match({\mathscr{P}},{\mathscr{C}},\theta )\}|$$

19 $${N}_{c}^{+}=|\{{C}_{j}|\,\exists \,{P}_{i},\,NA({P}_{i},{C}_{j})\ge \theta ,\,({P}_{i},{C}_{j})\in Match({\mathscr{P}},{\mathscr{C}},\theta )\}|$$

20 $$Precisio{n}^{+}=\frac{{N}_{p}^{+}}{|{\mathscr{P}}|}$$

21 $$Recal{l}^{+}=\frac{{N}_{c}^{+}}{|{\mathscr{C}}|}$$

22 $$F-measur{e}^{+}=\frac{2\times Precisio{n}^{+}\times Recal{l}^{+}}{Precisio{n}^{+}+Recal{l}^{+}}$$

In this definition, $Match({\mathscr{P}},{\mathscr{C}},\theta )$ contains the set of edges obtained by applying maximum non weighted bipartite graph matching algorithm on the bipartite graph that consists of edges between reference complexes and detected clusters that has the affinity score greater than *θ*. Since both *MMR* and *F* – *measure*^+^ are in range $[0,1]$, we can consider the sum of these two criteria as the benchmark for ranking protein complex detection methods.

**Figure 11:**
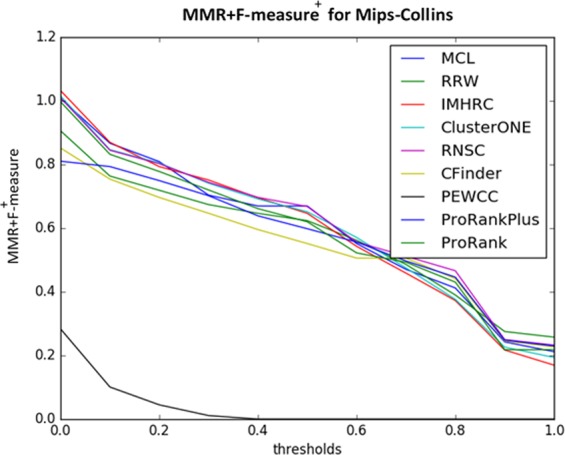
The values of the *MMR* + *Fmeasure*^+^ for all values of *θ*.

Obviously, the best algorithm for identifying protein complexes is an algorithm that has a one-to-one and spanning relationship between the detected clusters and the complexes within the gold standard. Therefore, this metric is suitable for evaluation, which can measure the quantity as well as the quality of this relation, while the old metrics do not have this property. The criterion introduced in this section explicitly examines the quality and quantity of such a relationship. It can be inferred that this criterion takes its maximum value 2 when the algorithm returns the clusters the same as the gold standard complexes. The values of this criterion for all methods applied on Collins dataset and compared with Mips gold standard is shown in Fig. 11 (ProRank is not considered in this comparison since it uses additional biological data).

This criterion still depends on the value of *θ*. Thus, for having a metric that is invariant with respect to *θ*, we consider the Area Under (*MMR* + *Fmeasure*^+^, *θ*) curve (*AUMF*) as a criterion for evaluating the results of the algorithms. The values of *AUMF* for 8 methods with default parameter values on each pair of dataset and gold standard are reported in Supplementary Table 1.

## Supplementary information

Supplementary File

LaTeX Supplementary File

## Data Availability

The package is implemented in Java and from the “CDAP” website (http://www.eslahchilab.ir/softwares/cdap)
