# Supplementary material for: CDAP: An Online Package for Evaluation of Complex Detection Methods
Source: Sci Rep. 2019 Sep 4;9:12751. doi: 10.1038/s41598-019-49225-7 (PMC6726630; doi:10.1038/s41598-019-49225-7)
Supplement: Supplementary file 1 — Supplementary File [file 41598_2019_49225_MOESM1_ESM.pdf]

CDAP: An Online Package for Evaluation of Complex Detection Methods

Ali M.A. Maddi[1], Fatemeh Ahmadi Moughari[2], Mohammad Mehdi Balouchi[2] , and Changiz Eslahchi[21*]

Gold Standard	MIPS				SGD			
Dataset	Collins	Gavin	Krogan-core	Krogan-Extnded	Collins2	Gavin3	Krogan-core4	Krogan-Extnded5
**Methods**								
AP	0.187	0.17	0.195	0.169	0.254	0.224	0.26	0.242
Cfinder	0.212	0.188	0.208	0.186	0.29	0.248	0.276	0.254
CMC	0.262	**0.233**	0.252	0.228	0.4	0.348	0.38	0.352
IMHRC	0.247	0.228	0.269	0.222	0.324	0.28	0.346	0.328
MCL	0.185	0.166	0.194	0.175	0.263	0.226	0.277	0.261
ClusterONE	0.174	0.155	0.184	0.159	0.248	0.207	0.264	0.242
RNSC	**0.293**	0.26	**0.287**	**0.253**	**0.433**	**0.371**	**0.444**	**0.405**
RRW	0.249	0.224	0.246	0.216	0.375	0.338	0.371	0.344

Supplementary Table 1. **Values of AUMF for every method on each pair of (gold standard, dataset):** The greatest AUMF on each (gold standard, dataset) is shown in bold face and underlined

Valid format for uploading results of custom algorithm:
The results of algorithm must be presented in a '.txt' file that in each line the STRING ID of proteins in one detected cluster are listed and separated by tab ('\t').
**Valid format for uploading results of custom PPI dataset:**
PPI dataset must be presented in a '.txt' file that in each line the STRING ID of two interacting proteins and the weights of their edge in PPI network are typed and separated by tab ('\t').
**Valid format for uploading results of custom Gold Standard:**
Gold Standard must be presented in a '.txt' file that in each line the STRING ID of proteins in one complex is listed and separated by tab ('\t').

Supplementary Table 2. **Valid formats for the uploaded files.**

[1] School of biological sciences, Institute for research in fundamental sciences(IPM), Tehran, 193955746, Iran
[2] Department of Computer Sciences, Faculty of Mathematics, Shahid Beheshti University, G.C. , Tehran, 1983963113, Iran

Guidelines for sending the codes of a new algorithm:
The codes should be executable by Linux terminal. It must give inputs from file and returns by writing in a file. The results of algorithm must be saved in a '.txt' file that in each line the STRING ID of proteins in one detected cluster are listed and separated by tab ('\t'). Using *sudoer* commands is not allowed. The codes must not contain the command that need User Interface (UI).
**Guidelines for sending a new PPI dataset:**
PPI dataset must be presented in a '.txt' file that in each line the STRING ID of two interacting proteins and the weights of their edge in PPI network are typed and separated by tab ('\t').
**Guidelines for sending a new Gold Standard:**
Gold Standard must be presented in a '.txt' file that in each line the STRING ID of proteins in one complex is listed and separated by tab ('\t').

Supplementary Table 3. **Guidelines for sending files for extending CDAP.**

Reference	Complex
**YRC**	YLR305C YGR198W
**YRC**	YIL062C YPR093C YER048C YDL017W YLR429W YNL106C YEL001C YNL073W YOR3513C YOR145C YDR032C YDR314C YDR129C YPL218W YIL105C YDR212W YIL039W YGR198W
**YRC**	YLR175W YHL038C YGR150C YOL139C YHR052W YKR024C YGR145W YCL011C YCL11C YMR290C YDR174W YBL002W YBL0104 YAR073W YLR432W YLL027W YNL132W YNL308C YJR144W YDR194C YOR206W YOX001 YOL041C YDL213C YDR432W YOR017W YEL055C YGL120C YDR496C YLR196W YDR091C YPL012W YMR229C
**Paper**	YMR212C YLR305C YGR198W
**DIP**	YLR175W YDR091C YGR150C YHR099W YDR174W YER006W YOL139C YJL109C YOL041C YGL120C YGR162W YLR432W YLL027W YCL059C YCL59C YBL002W YBL0104 YDR432W YKR081C YKR401 YNL132W YPR016C YAR073W YBL004W YBL0101 YOR206W YOX001 YMR049C YJR144W YDR496C YDR381W YDR224C YOR272W YGR145W YHL038C YGR198W YLR196W YOR017W YPL012W YMR229C YM9959.11C YDL060W YPH1 YGR103W YLL008 YGL068W YKR024C YEL055C YOR310C YMR290C YDL213C YNA1 YNL061W YNL2428W YNL308C YCL011C YCL11C YJR041C YKR059W YJL138C YDR194C YD9346.05C YLR347C YHR052W YKL014C
**Links of datatbases**	
**YRC**	http://www.yeastrc.org/pdr/yeastProteinRedirect.do?acc=YGR198W#msData
**DIP**	https://dip.doe-mbi.ucla.edu/dip/Browse.cgi?PK=6350&MD=1
**Paper**	http://jcb.rupress.org/content/jcb/183/6/1061.full.pdf

Supplementary Table 4. **Reported Complexes including YGR198W in known databases.**

GoldSatndard-Dataset	Method	Detected Cluster
MIPS-KroganCore	RNSC	YGR198W YLR380W
	MCL	YIL105C YLR429W YNL106C YDR129C YGR198W YDL017W YDR314C YEL001C YER048C YOR145C YPL218W YNL073W YPR093C YDR032C YIL039W
	Cfinder	YGR198W YCR058C YJL069C YLR129W YLR222C YLR409C YNR054C YDR449C
MIPS-KroganExtended	RNSC	YGR198W YDR212W YCL064C
	MCL	YIL105C YLR429W YNL106C YDR129C YGR080W YGR198W YDL017W YER048C GL162W YLR142W YEL001C YNL208W YDL210W YPL218W YKL105C YOR145C YPL260W YNL073W YDR032C YPR093C YDR063W YIL039W
SGD-KroganCore	RNSC	YGR198W YEL051W
	Cfinder	YGR198W YCR058C YJL069C YLR129W YLR222C YLR409C YNR054C YDR449C
SGD-KroganExtended	MCL	YGR198W YDL210W

Supplementary Table 5. **Clusters detected by each method on each of dataset and gold standard pairs including YGR198W.**
